# Intestinal Mucosal Barrier Is Injured by BMP2/4 via Activation of NF-**κ**B Signals after Ischemic Reperfusion

**DOI:** 10.1155/2014/901530

**Published:** 2014-07-16

**Authors:** Kang Chen, Wei Xie, Binyu Luo, Weidong Xiao, Daniel H. Teitelbaum, Hua Yang, Kebin Zhang, Chaojun Zhang

**Affiliations:** ^1^Department of General Surgery, Xinqiao Hospital, Third Military Medical University, Chongqing 400037, China; ^2^Center of Medical Experiment & Technology, Xinqiao Hospital, Third Military Medical University, Chongqing 400037, China; ^3^Department of Surgery, University of Michigan, Ann Arbor, MI 48109, USA

## Abstract

Intestinal ischemic reperfusion (I/R) can cause dysfunction of the intestinal mucosal barrier; however, the mechanism of the intestinal mucosal barrier dysfunction caused by I/R remains unclear. In this study, using intestinal epithelial cells under anaerobic cultivation and an in vivo rat intestinal I/R model, we found that hypoxia and I/R increased the expression of BMP2/4 and upregulated BMP type Ia receptor and BMP type II receptor expression. We also found that exogenous BMP2/4 can activate the ERK and AKT signaling pathways in rat small intestine (IEC-6) cells, thereby activating NF-*κ*B signaling, which leads to increased levels of inflammatory factors, such as TNF-*α* and IL-6. Furthermore, recombinant BMP2/4 decreased the expression of the tight junction protein occludin via the activation of the NF-*κ*B pathway; these effects were abolished by treatment with the BMP-specific antagonist noggin or the NF-*κ*B inhibitor pyrrolidine dithiocarbamate (PDTC). All these factors can destroy the intestinal mucosal barrier, thereby leading to weaker barrier function. On the basis of these data, we conclude that BMP2/4 may act as the pathogenic basis for intestinal mucosal barrier dysfunction when the intestines suffer an I/R injury. Our results provide background for the development pharmacologic interventions in the management of I/R injury.

## 1. Introduction

Intestinal ischemic reperfusion (I/R) injury is a common pathophysiological process and can be caused by major vascular surgery, mesenteric artery occlusion, small bowel transplantation, cardiopulmonary bypass, hemodialysis, strangulated hernias, trauma, and shock [1, 2]. Acute intestinal ischemia is a life-threatening vascular emergency; however, the improvement in recent years has been minimal, and its in-hospital mortality rate remains high at approximately 60%–80% [3, 4]. Although many studies have focused on I/R, the mechanisms have not been fully elucidated. Many cytokines, such as TNF-*α*, IL-6, IL-1, and ICAM-1, have been reported to be involved in the process of intestinal I/R injury, and their expression can be regulated by nuclear factor-kappa B (NF-*κ*B) [5–7]. These cytokines may increase capillary permeability and damage the intestinal microcirculation, which in turn may cause the intestinal mucosal membrane to lose its resistance to bacteria. This results in bacterial translocation and endotoxemia, ultimately leading to the systemic inflammatory response syndrome (SIRS) and multiple organ dysfunction (MODF). It has been well demonstrated that the NF-*κ*B pathway plays an important role in this cytokine-induced intestinal barrier dysfunction [8, 9]. The application of NF-*κ*B inhibitors was found to significantly attenuate the expression of inflammatory cytokines and to alleviate intestinal I/R injury [10]. Therefore, understanding the NF-*κ*B activation mechanism will provide new means for the clinical treatment of intestinal I/R injury and reduce the mortality of severe I/R-injured patients.

Bone morphogenic protein (BMP) is a key member of the transforming growth factor- (TGF-) *β* super family. More than 30 BMP proteins have been identified, and they can be further classified into several subgroups, including the BMP2/4 group, the BMP5/6/7/8 group (OP-1 [osteogenic protein-1] group), the growth and differentiation factor- (GDF-) 5/6/7 group, and the BMP9/10 group [11]. The BMP2/4 and BMP5/6/7 groups are the best-characterized members of this family. After these receptors combine with their ligands, the BMP proteins function via either typical or atypical pathways. The canonical pathway activates the phosphorylation of Smad 1, 5, and 8, while the noncanonical pathway activates the NF-*κ*B pathway [12]. Our previous studies have shown that the expression of BMP2/4 in intestinal epithelial cells increased in TPN mice, the ERK1/2 pathway was activated, and the proliferation of intestinal epithelial cells was weakened [13]. These effects may lead to weaker intestinal barrier function and thereby increase the possibility of endogenous infection. BMPs have recently been thought to influence inflammatory processes in adults due to their chemotactic activity on fibroblasts, myocytes, and inflammatory cells [14]. It has been shown that BMP2 can induce inflammatory reactions in endothelial cells, fibroblasts, preosteoblasts, and soft tissues. Sorescu et al. [15] found that BMP4 plays an inflammatory role in the early steps of atherogenesis by initiating an inflammatory cascade in an NF-*κ*B-dependent manner through the stimulation of ICAM-1 surface expression in activated endothelial cells. The activation of NF-*κ*B in human embryonic kidney (HEK) cells also depends on the BMP signaling pathway [12]. Therefore, we hypothesized that the BMP pathway would be upregulated and play an important role, through activation of the NF-*κ*B signaling pathway, in the damage caused to the intestinal barrier function by I/R injury. Our data indicate that BMP2/4 expression is increased in both an early cell hypoxia model and a male Sprague-Dawley (SD) rat I/R model. BMP2/4 is able to directly increase the expression of the cytokines TNF-*α* and IL-6 and decrease the expression of the tight junction protein occludin by activating NF-*κ*B signaling. This effect was attenuated in part by the BMP-specific antagonist noggin.

## 2. Materials and Methods

Anti-p-p65, anti-p65, anti-p-ERK1/2, anti-ERK1/2, anti-p-AKT, and anti-AKT were purchased from Cell Signaling Technology (Boston, MA, USA). Anti-GAPDH antibody was purchased from the ProteinTech Group (Chicago, IL, USA). Anti-BMP2, anti-BMP4, anti-BMPRIa, anti-BMPRII, anti-BMPRIb, and anti-occludin antibodies were purchased from Santa Cruz Biotechnology (Santa Cruz, CA, USA). Recombinant BMP2, BMP4, and noggin were purchased from Peprotech (NJ, USA). The inhibitor of NF-*κ*B, PDTC, was purchased from Beyotime (Wuhan, China).

### 2.1. Cell Culture

IEC-6 intestinal epithelial cells were purchased from the American Type Culture Collection (ATCC, Manassas, VA) and grown in Dulbecco's modified Eagle's medium (DMEM, Hyclone) supplemented with 10% fetal bovine serum (Gibco), 100 IU/mL penicillin, and 100 mg/mL streptomycin. The IEC-6 cells were cultured at 37°C in either normoxic (5% CO_2_ and 20% O_2_) or hypoxic (5% CO_2_ and 1% O_2_ in a hypoxia chamber) conditions (Thermo Fisher Scientific, Ohio, USA). For Western blot analysis, BMP2, BMP4, and noggin were added to the medium for 6 h or for a time gradient from 0 min to 120 min. For real-time PCR analysis, BMP2, BMP4, and noggin were added to the medium for 3 h. For immunofluorescence analysis, BMP2, BMP4, and noggin were added to the medium for 30 min.

### 2.2. Western Blot Analysis

The cells were washed twice with phosphate-buffered saline (PBS) before lysis in cold RIPA buffer (50 mM Tris, 150 mM NaCl, 1% Triton X-100, 1% sodium deoxycholate, 0.1% SDS, and 2 mM sodium pyrophosphate).

Samples were mixed with loading buffer and boiled for 5 min before electrophoresis. Proteins were loaded onto 8–10% SDS-PAGE gels at 100 V for 2 h. After electrophoresis, the proteins were electroblotted onto NC membranes at 200 mA for 2 h. Nonspecific binding was blocked by incubation in Tris-buffered saline (TBS) containing 0.1% Tween 20 (TBS-T) and 5% skim milk. The transferred membranes were incubated overnight at 4°C with primary antibody as follows: anti-p-p65 (1 : 1000), anti-p65 (1 : 1000), anti-p-ERK (1 : 500), anti-ERK (1 : 500), anti-p-AKT (1 : 1000), anti-AKT (1 : 1000), anti-BMP2 (1 : 500), anti-BMP4 (1 : 500), anti-BMPRIa (1 : 200), anti-BMPRII (1 : 750), anti-BMPRIb (1 : 200), and anti-GAPDH (1 : 5000). After washing three times in TBS-T, the membranes were incubated with anti-rabbit IgG (Zhongshan Bio., China) conjugated to horseradish peroxidase at a dilution of 1 : 3000 in TBS-T containing 5% skim milk for 1 h at 37 °C. After three additional washes with TBS-T, the signals were visualized using the Super Signal West Pico trial kit (Pierce, USA) and detected with Image Station 4000R (Kodak).

### 2.3. Animal Experiments

Male Sprague-Dawley (SD) rats weighing 200–250 g were obtained from our university's Laboratory Animals. After the rats were fasted for 12 hours, their abdomens were opened via a midline incision under sodium pentobarbital anesthesia. The rats were randomly assigned to three groups: (1) the I/R group, in which the superior mesenteric artery (SMA) was occluded for 30 minutes followed by defined times of reperfusion; (2) the noggin+ I/R group, in which an intraperitoneal injection of 4 *μ*g/kg noggin was given 30 min before I/R, followed by ischemia, reperfusion, and sacrifice of the rats at defined times; and (3) the sham group, which included animals subjected to anesthesia and laparotomy without ischemia. The tissue was cut along the longitudinal axis, washed in physiological saline, and immediately frozen in liquid nitrogen and stored at −70°C for future use. All animal experiments were performed in compliance with our university's Guidelines for the Care and Use of Laboratory Animals.

### 2.4. Immunofluorescence Analysis

For the current study, 10 *μ*m frozen sections were cut from the jejunum and fixed on slides. After fixation in 4% formaldehyde for 20 min, the sections were incubated in 3% H_2_O_2_ for 30 min to quench endogenous peroxidases. Nonspecific binding was blocked with 5% BSA in phosphate buffered saline for 30 min at room temperature. Sections were incubated overnight at 4°C in 3% BSA in PBS with primary antibodies as follows: anti-BMP2, anti-BMP4, anti-BMPRIa, anti-BMPR2 (Santa Cruz Biotechnology, Inc., Santa Cruz, CA, USA) in a 1 : 50 dilution, and anti-P65 (Cell Signaling Biotechnology) in a 1 : 100 dilution. Sections were washed three times in BSA in PBS and incubated with Alexa 488-conjugated goat anti-rabbit antibody for 1 h at RT. After the nuclei were stained with DAPI, images were analyzed and collected with a Leica TCSSP confocal imaging system (Leica, Heidelberg, Germany).

The expression of NF-*κ*B in IEC-6 cells was also assayed with an immunofluorescent technique. IEC-6 monolayers were washed three times with PBS before fixation in 4% paraformaldehyde for 20 min at room temperature. After another three washes, the IEC-6 cells were permeabilized with 0.2% Triton X-100 for 10 min and blocked in 5% BSA in PBS at room temperature for 30 min. Then, the IEC-6 cells were incubated overnight at 4°C in 5% BSA in PBS with anti-p65 at a 1 : 50 dilution. Monolayers were washed with PBS and incubated with FITC green-conjugated goat anti-rabbit secondary antibodies (1 : 100; Zhongshan) for 1 h in dark conditions. DAPI (Sigma-Aldrich, St. Louis, MO) was used to stain the nuclei, and the cells were imaged using a laser scanning fluorescence microscope (Leica, Heidelberg, Germany).

### 2.5. Real-Time PCR Analysis

Cells were washed two to four times with PBS prior to RNA isolation. Total cellular RNA was isolated with Trizol reagent and used for first strand cDNA synthesis with the Reverse Transcription System. Quantification of gene transcripts was performed with a 7500 Real-Time PCR System (Applied Biosystems, Foster City, California, USA) using Power SYBR Green (Applied Biosystems, Foster City, California, USA). PCR conditions were one cycle of 94°C for 2 min followed by 50 cycles of 94°C for 10 s, a specified annealing temperature for 15 s and 72°C for 15 s. Amplification was followed by melting curve analysis, which used the following program: one cycle at 65°C for 1 s, 94°C for 2 s, and 37°C for 5 s. The housekeeping gene *β*-actin was used as an endogenous reference gene to which the expression of the other genes was normalized using the comparative cycle of threshold value. The following PCR primers were used: TNF-a forward primer: 5′-GCGTGTTCATCCGTTCTCTA-3′, reverse primer 5′-CGTCTCGTGTGTTTCTGAGC-3′; IL-6 forward primer: 5′-AGTTGCCTTCTTGGGACTGA-3′, reverse primer: 5′-ACTGGTCTGTTGTGGGTGGT-3′; and *β*-actin forward primer: 5′-CCCATCTATGAGGGTTACGC-3′, reverse primer: 5′-TTTAATGTCACGCACGATTTC-3′.

### 2.6. Statistical Analyses

Statistical analyses were performed using SPSS 13.0 software. All experimental data are shown as means ± SD. Comparisons among 3 or more groups were made by analysis of variance (ANOVA), and 2 groups were compared by Student's *t*-test. A *P* value less than 0.05 was considered statistically significant in all cases. All reported significance levels represent 2-tailed *P* values. If not otherwise stated, all experiments were repeated for at least 3 individual experiments to ensure reproducibility.

## 3. Results

### 3.1. Hypoxia and I/R Induced the Expression of BMP2 and BMP4 in Intestinal Epithelial Cells

**Figure 1:**
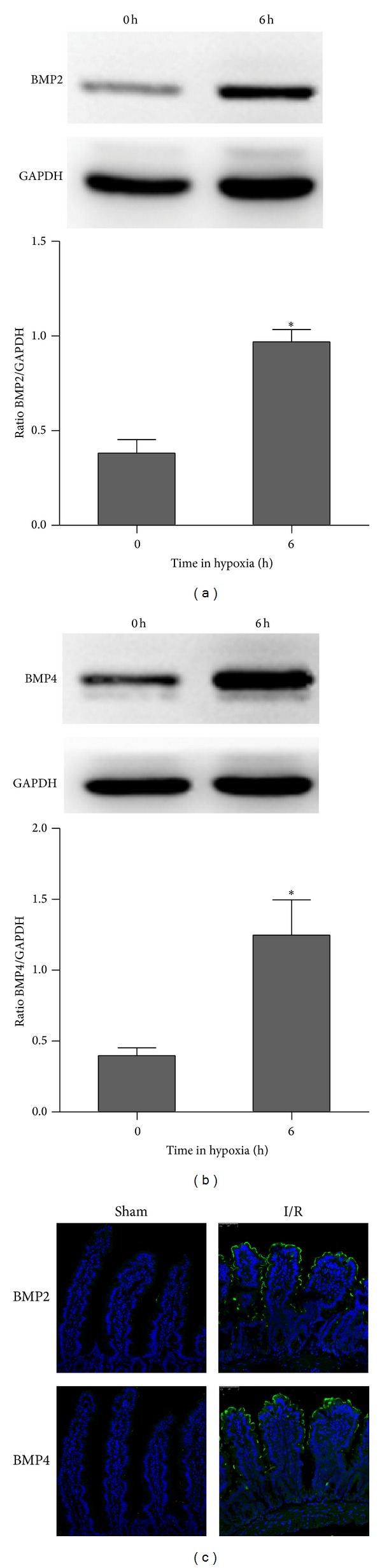
The expression of BMP2 and BMP4 in intestinal epithelial cells. (a) and (b) The IEC-6 cells were treated with hypoxia (1% O_2_) for 6 h. Hypoxia caused a dramatic increase in BMP2 and BMP4 protein expression as detected by Western blotting. *P < 0.05 versus control. Data are representative of 3 similar experiments. (c) The level of BMP2 protein expression significantly increased in the mid-to-distal villus region after 1 h of I/R, while the level of BMP4 protein expression also significantly increased in both the villi and mesenchyme.

We analyzed the protein level of BMP2 and BMP4 with Western blotting. We found that the expression level of BMP2 and BMP4 was upregulated 2.5-fold (Figure 1(a)) and 3.1-fold (Figure 1(b)), respectively, in IEC-6 cells after 6 h of hypoxia. Meanwhile, we detected the expression of BMP2 and BMP4 in intestinal epithelial cells in an I/R rat model. IF analysis showed that these proteins were also significantly increased along the crypt/villus axis after 1 h of I/R, consistent with the significantly increased BMP2 and BMP4 levels in intestinal epithelial cells under hypoxia. Normally, BMP2 and BMP4 are expressed in both the epithelial and mesenchymal compartments, but BMP4 is highly expressed and enriched in the mesenchyme [13, 16]. In the present study, the BMP2 level significantly increased in the mid-to-distal villus region after 1 h of I/R, while the BMP4 level increased significantly in both the villi and mesenchyme in the I/R rat (Figure 1(c)).

### 3.2. BMP Receptor (BMPRIa and BMPRII) Expression Levels Were Upregulated with Hypoxia and I/R

**Figure 2:**
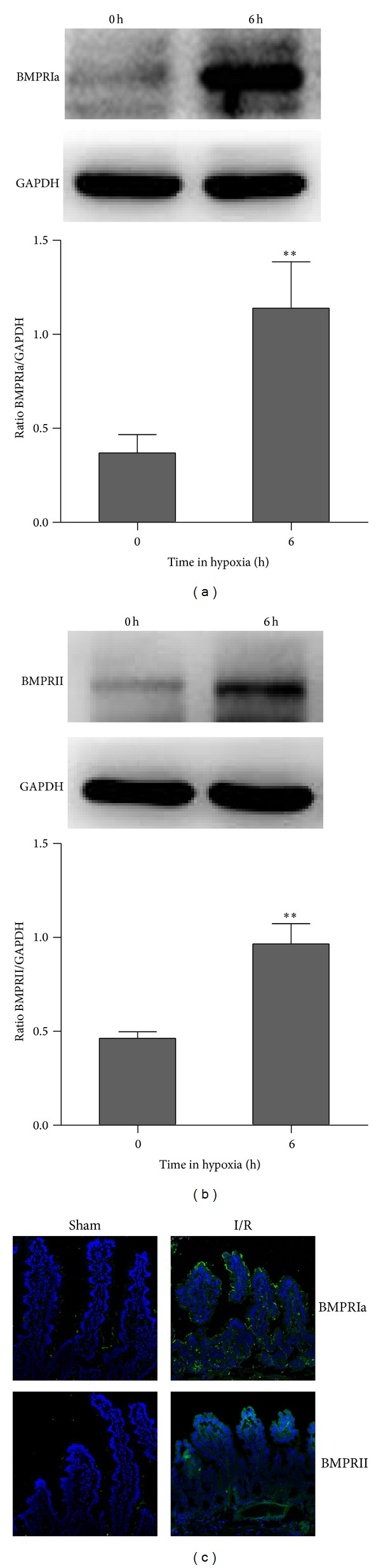
(a) and (b), (c) BMPRIa and BMPRII expression was detected by Western blotting and immunofluorescence staining. BMPRIa and BMPRII expression levels were both significantly increased after 6 h of hypoxia in IEC-6 cells. **P < 0.01 versus control. BMPRIa and BMPRII expression in the intestinal mucosa also increased after I/R for 1 h compared to the control. Data are representative of 3 similar experiments.

The main BMP receptors include the type II BMP receptor (BMPRII) and the following type I receptors: the BMPRI group (BMPRIa and BMPRIb; also denoted as ALK-3 and ALK-6, resp.), the ALK-1 group (ALK-1 and ALK-2), and the TbR-I group (ALK-4/ActR-IB, ALK-5/TbR-I, and ALK-7). Typically, BMP2 and BMP4 bind to BMPRIa and BMPRIb, but BMPRIa has a high-affinity binding site for BMP2 [11]. To investigate whether the greater abundance of BMP2/4 led to an increase in intracellular BMP signaling, we evaluated the expression of BMPRII and BMPR-Ia in epithelial cells under hypoxia and I/R. At 6 h after hypoxia, BMPRIa and BMPRII expression levels were both significantly increased (Figures 2(a) and 2(b)). We also detected the expression of BMP receptors in the rat I/R model. The rats were euthanized after 1 h of I/R treatment. Sections of the small intestine were collected to detect changes in BMPRIa and BMPRII expression via immunofluorescence analysis. Immunofluorescence staining showed that the expression levels of the transmembrane receptors BMPRIa and BMPRII were significantly increased in the villi but had lower expression levels in the matrix (Figure 2(c)).

### 3.3. Exogenous BMP2 and BMP4 Activated the NF-*κ*B Pathway

**Figure 3:**
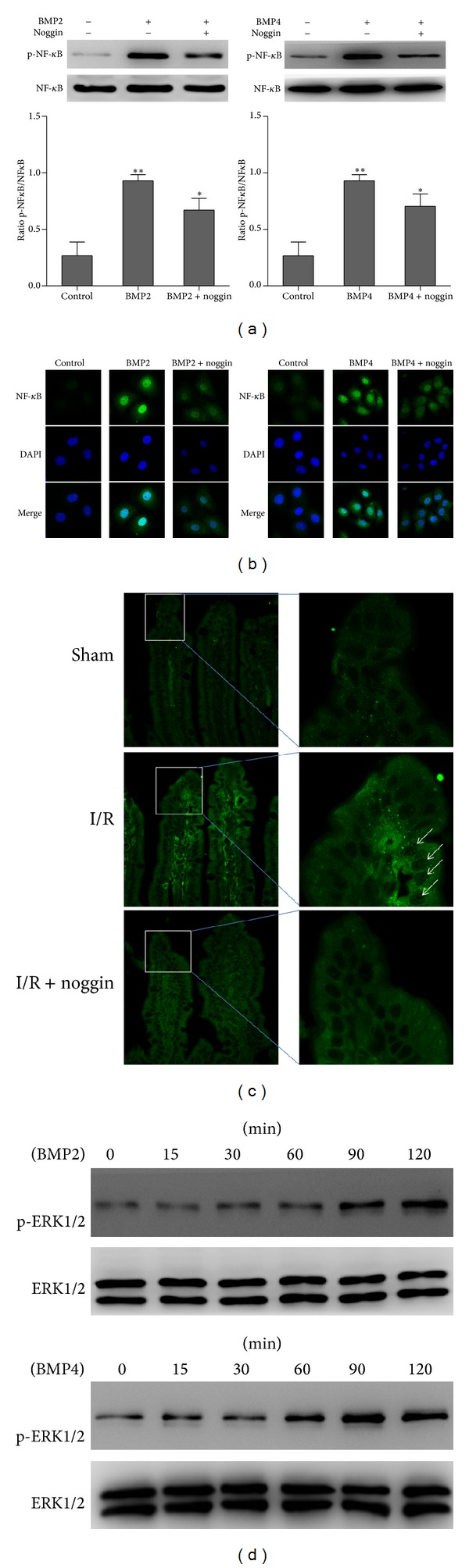
(a) Western blotting determined the expression of phosphorylated NF-*κ*B in IEC-6 after treatment with BMP2 and BMP4 for 6 h. The phosphorylated NF-*κ*B significantly increased compared with the control group, **P < 0.01 versus control. Noggin partially decreased NF-*κ*B transcriptional activity. *P < 0.05, different from a single treatment with BMP2 or BMP4. (b) Immunofluorescence detected the translocation of NF-*κ*B to the nucleus after treatment with BMP2 and BMP4 for 30 min, and noggin partially reversed the nuclear localization of NF-*κ*B. (c) The fluorescence intensity of phosphorylated NF-*κ*B was significantly increased in the I/R group compared to the sham group. In the I/R group, NF-*κ*B exhibited significant nuclear localization in the distal villus, where abundant BMP2 and BMP4 are secreted after I/R (as shown in Figure 1(c)). The abundant BMP2 and BMP4 directly activated NF-*κ*B resulting in its nuclear localization, and noggin decreased the nuclear localization of NF-*κ*B in the I/R + noggin group. (d) Western blotting detected ERK expression upon BMP2 and BMP4 treatment at the defined time points. Phosphorylated ERK1/2 expression progressively increased in a time-dependent manner.

We used Western blotting to determine the effect of BMP2 and BMP4 on NF-*κ*B transcriptional activity. BMP2 and BMP4 increased NF-*κ*B transcriptional activity 3.5-fold and 3.4-fold, respectively, while BMP2/4 combined with noggin resulted in lower levels of NF-*κ*B transcriptional activity (Figure 3(a)). NF-*κ*B is normally sequestered in the cytoplasm. Once activated, NF-*κ*B translates to the nucleus to trigger the transcription of genes involved in inflammatory cellular responses and other types of signals. We used IF to detect the expression of NF-*κ*B after stimulation by exogenous BMP2/4 for 30 min. The fluorescence intensity was greater compared to the control group, and blockade of Bmp2/4 signaling by noggin completely reversed the nuclear localization of NF-*κ*B induced by BMP2 and BMP4 (Figure 3(b)). Meanwhile, the in vivo re**s**ults showed that NF-*κ*B signaling was obviously activated in intestinal epithelial cells after 1 h of I/R treatment. In contrast, after intraperitoneal injection of 4 *μ*g/kg noggin 30 min before I/R, the expression of NF-*κ*B was significantly inhibited (Figure 3(c)). Because mitogen-activated protein kinase (MAPK) is known as an upstream regulator of NF-*κ*B [17], we evaluated the three common proteins of the MAPK pathway, ERK, P38, and JNK. Exogenous BMP2 and BMP4 activated ERK (Figure 3(d)) but not P38 or JNK (data not shown). These results are consistent with the results from S. O. Kim and M. R. Kim [17], who found that treatment with an ERK-specific blocking agent completely inhibited NF-*κ*B activity. These results indicate that NF-*κ*B may be activated by BMP2 and BMP4 via an increase in ERK phosphorylation.

### 3.4. The Expression of the Inflammatory Cytokines TNF-*α* and IL-6 Induced by BMP2 and BMP4 in Intestinal Epithelial Cells

**Figure 4:**
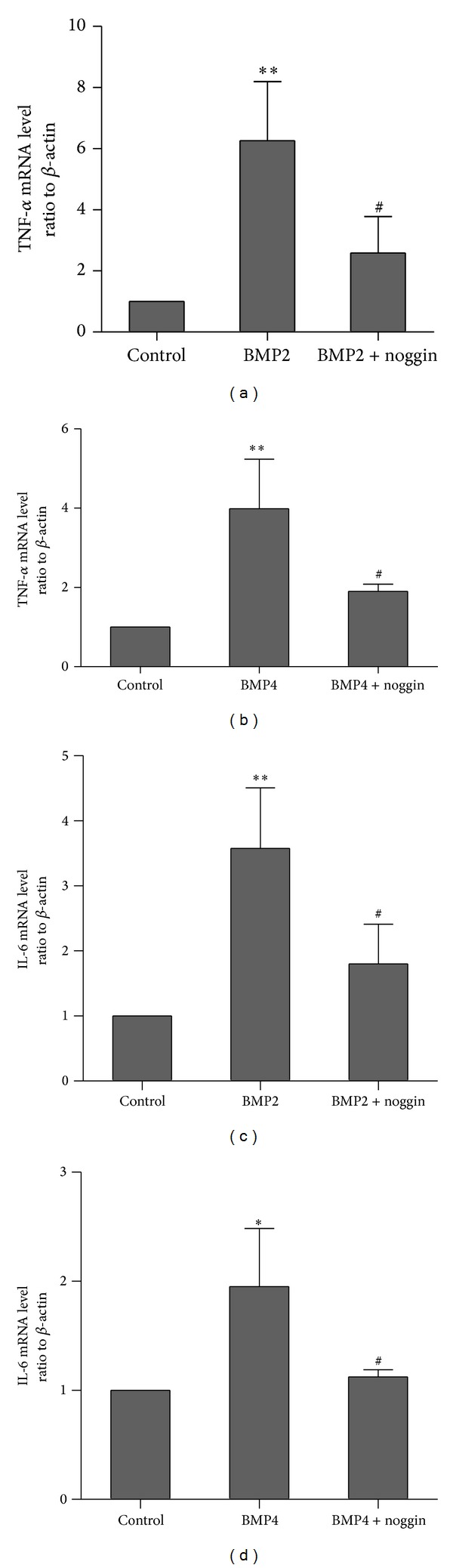
(a) BMP2 (100 ng/mL) significantly induced the expression of TNF-*α* mRNA. BMP2 combined with noggin partially reversed the increase in the TNF-*α* mRNA. (b) The effect of BMP4 in inducing the expression of TNF-*α* mRNA was weaker compared to BMP2, and the expression of TNF-*α* mRNA induced by BMP4 was also decreased by noggin. (c) and (d) BMP4 and BMP2 both increased the expression of IL-6 mRNA, and these effects were decreased by noggin. *Different from the control after BMP2 and BMP4 treatment, **P < 0.01, *P < 0.05. ^#^Different from a single treatment with BMP2 or BMP4, *P* < 0.05. Data are representative of 3 similar experiments.

NF-*κ*B plays a central role in regulating the transcription of cytokines, adhesion molecules, and other mediators involved in acute respiratory distress syndrome (ARDS), sepsis, and multiple organ dysfunction syndrome (MODS) [6]. To test whether the activation of NF-*κ*B induced by BMP2 and BMP4 resulted in an increase in inflammatory cytokines, we used RT-PCR to detect the expression of TNF-*α* mRNA and IL-6 mRNA in IEC-6 cells after treatment with BMP2 and BMP4 for 3 h. Treatment of IEC-6 cells with 100 ng/mL BMP2 caused the level of TNF-*α* mRNA to increase 6.3-fold compared to the control group (Figure 4(a)), while the effect of BMP4 in inducing the expression of TNF-*α* mRNA was weaker (Figure 4(b)). These effects were decreased by noggin. Tumor necrosis factor is one of the most powerful inducers and promoters of inflammation [3], and NF-*κ*B both is activated by cytokines and induces the expression of inflammatory cytokines. This gives rise to the potential for NF-*κ*B activation to spread from cell to cell within a tissue and beyond [18]. We also evaluated the expression of IL-6 mRNA. BMP2 and BMP4 both increased the expression of IL-6 mRNA, and these effects were decreased by noggin (Figures 4(c) and 4(d)).

### 3.5. BMP2 and BMP4 Disrupted Tight Junctions via the Activation of the NF-*κ*B Pathway

**Figure 5:**
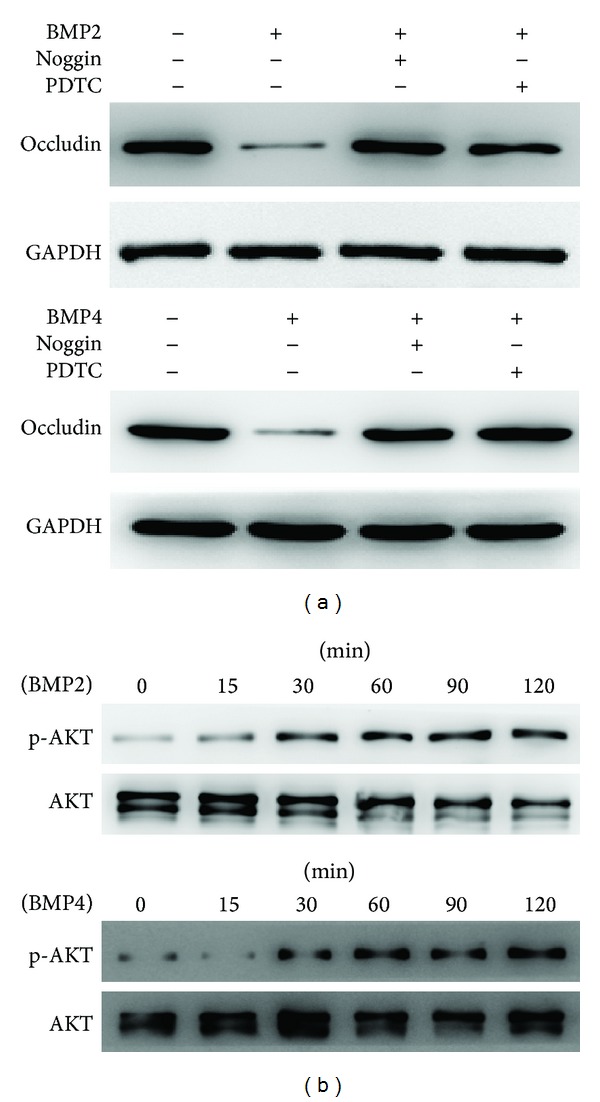
(a) Occludin protein expression was decreased in IEC-6 cells upon BMP2 or BMP4 treatment for 24 h, but both noggin and PDTC reversed the decrease in occludin expression in IEC-6 cells. (b) AKT phosphorylation progressively increased from 15 min to 120 min with BMP2 or BMP4 treatment.

The activation of NF-*κ*B by IFN-*γ* enhances the permeability of T84 cells and decreases the levels of intercellular tight junction proteins, whereas the inhibition of NF-*κ*B will block the increase in T84TJ permeability and alter occludin expression [8, 19]. We asked whether BMP2/4 would disrupt the intestinal mucosal barrier function via the activation of NF-*κ*B. We used recombinant BMP2 and BMP4 to stimulate intestinal epithelial cells for 24 h, and the tight junction protein occludin was detected by Western blotting. The expression of occludin decreased after treatment with BMP2 and BMP4, while this effect was abolished by noggin or the NF-*κ*B inhibitor PDTC (Figure 5(a)). These results may indicate that BMP2 and BMP4 disrupt the integrity of the intestinal mucosal barrier via the activation of NF-*κ*B and that PDTC can reverse the decrease in TJ proteins. Boivin et al. have shown that PI3-K/Akt activation was required for the activation of NF-*κ*B pathways in the modulation of the TJ barrier by treatment with IFN-*γ* [8]. We also detected the expression of AKT at the indicated times after stimulation of the IEC-6 cells with BMP2 and BMP4. The phosphorylation of AKT progressively increased in a time-dependent manner (Figure 5(b)).

## 4. Discussion 

BMP belongs to the TGF-*β* super family. Previous research has focused on the roles of the BMP pathway in early intestinal development and in the proliferation and differentiation of intestinal epithelial cells [20–22]. However, the relationship between the BMP pathway and intestinal mucosal barrier dysfunction caused by I/R has rarely been examined. Shen et al. [23] found that, in rats after acetabular surgery, treatment with 4 mg/mL BMP2 protein significantly induced local inflammation, including an early and pronounced polymorphonuclear cell infiltration accompanied by the increased expression of TNF-*α* and IL-6. Intestinal I/R results in the release of abundant inflammatory factors, the generation of oxygen free radicals (ROS), and the activation of NF-*κ*B. These factors lead to enhanced permeability in the intestinal barrier and systemic inflammatory reactions [24, 25].

Because of the critical role of BMP in inflammatory reactions, we hypothesized that the BMP signaling pathway would contribute to the mechanisms involved in promoting I/R-associated intestinal mucosal barrier injury. Our present study shows that, with hypoxia and I/R, intestinal epithelial cells produce abundant BMP2 and BMP4 and that BMPRIa and BMPRII expressions are also enhanced. Western blotting and IF showed that recombinant BMP2 and BMP4 directly activate NF-*κ*B in IEC-6 cells. BMP2/4 is able to directly increase the expression of the cytokines TNF-*α* and IL-6 and decrease the expression of the tight junction protein occludin by activating NF-*κ*B signaling. This effect was attenuated in part by either the BMP-specific antagonist noggin or the NF-*κ*B inhibitor PDTC. Our study may provide new evidence that the activation of NF-*κ*B in intestinal I/R injury involves the BMP signaling pathway.

It is interesting to note that BMP signaling is complex; different BMP subgroups can mediate antagonistic effects, or the same ligand can produce different effects in similar tissues. For example, in an in vivo model, BMP4 inhibits liver proliferation, and the BMP4 antagonist noggin enhances regeneration [26], whereas another report demonstrates that BMP7 is an endocrine factor expressed in the kidney that enhances liver regeneration [27]. Our findings demonstrate that hypoxia and I/R increased intestinal epithelial cell BMP2/4 signaling, thereby activating NF-*κ*B signaling. These changes led to increased expression of inflammatory factors, such as TNF-*α* and IL-6, and decreased expression of the tight junction protein occludin, which could result in disruption of the intestinal barrier. Our results are consistent with those of Masterson et al. [28] that, after 6 h in injured airways, BMP signaling is activated, E-cadherin expression is downregulated, and migration in normal adult airway epithelial cells is increased. Furthermore, inhibition of BMP activity protects epithelial barrier function in cases of lung injury [29].

However, our results contrast with another report that BMP7 has protective and anti-inflammatory functions in acute ischemic renal injury [30, 31]. In addition, BMP7 administration conferred intestinal mucosal protection and reduced systemic IL-6 expression levels in an inflammatory bowel disease model [32]. Additionally, BMP7 administration before intestinal I/R injury protects against intestinal mucosal injury and liver injury, preserves intestinal function, and prevents intestinal inflammation [33]. One possible explanation is that BMP7 preferentially acts through Alk2 (a stimulatory pathway), whereas BMP2/4 preferentially acts through Alk3 (an inhibitory pathway). Another possibility is that different doses of BMPs could activate different pathways. Alternative signal transduction mechanisms may also play a role: the canonical BMP pathway regulates gene expression via the SMAD-dependent pathway, while the noncanonical BMP pathway regulates NF-*κ*B via the MEK/ERK pathway. Our studies have demonstrated that phosphorylated ERK1/2 and AKT expression levels progressively increased in intestinal epithelial cells upon treatment with BMP2/4. Thus, we believe that NF-*κ*B may be activated by BMP2 and BMP4 via the MEK/ERK pathway in the hypoxia and intestinal I/R model.

Previous studies have shown that NF-*κ*B is activated in the process of intestinal I/R injury and that the amount of activated NF-*κ*B correlates with the degree of mucosal inflammation [34, 35]. The activation of NF-*κ*B could regulate the expression of several inflammatory genes, such as cell factors, adhesion molecules, and enzymes. In turn, the released inflammatory factors (e.g., TNF-*α* and IL-6) can activate NF-*κ*B, thereby aggravating the deterioration of intestinal barrier function. Additionally, the upregulated expression of TNF-*α* and IL-6 induced in intestinal epithelial cells by BMP2 and BMP4 treatment confirms that BMP2 and BMP4 can mediate the intestinal mucosal injury caused by I/R via the activation of NF-*κ*B. Moreover, BMP2 and BMP4 have been shown to activate ERK1/2, while the activation of the ERK pathway reduces the proliferation of intestinal epithelial cells [13]; thus, persistent BMP expression is unfavorable for the repair of intestinal mucosal injury. In addition to the amount of proinflammatory cytokines released by I/R, ischemia and hypoxia also disrupt the integrity of the intestinal mucosal barrier. We asked whether BMP2 and BMP4 could decrease the expression of tight junction proteins, and we evaluated changes in the expression of the tight junction protein occludin. Our results show that the expression of occludin decreased after treatment with BMP2 and BMP4, while these effects were abolished by treatment with noggin or PDTC. BMP2/4 can induce intestinal mucosal barrier dysfunction via the activation of NF-*κ*B. These results are consistent with those of Boivin et al., who used NF-*κ*B blockers to prevent the IFN*γ*-induced increase in epithelial permeability and the destruction of tight junction proteins [8]. Our previous study also found that IFN-*γ* induced intestinal barrier function injury via the NF-*κ*B/HIF-1a pathway [19].

There are many types of extracellular BMP antagonists, such as noggin, chordin, cerberus, and follistatin [36, 37]. By binding BMP, these secretory factors act as competitive antagonists and are important negative regulatory factors in the BMP pathway. In mouse intestine, transient expression of the BMP antagonist noggin has been observed in both pericryptal mesenchymal cells and intestinal epithelial stem cells, which may contribute to maintaining intestinal stem cell self-renewal by activating Wnt signaling and inhibiting BMP signaling of the basal crypt epithelial cells [38]. Our data indicate that BMP2/4 expression increased in both an early intestinal epithelial cell hypoxia model and a rat I/R model. Furthermore, NF-*κ*B was directly activated by recombinant BMP2/4 in IEC-6 cells by Western blotting and immunofluorescence (IF). Finally, BMP2/4 induced the expression of intracellular TNF-*α* mRNA and IL-6 mRNA in IEC-6 cells, and this effect was abolished by the BMP-specific antagonist noggin. The in vivo rat model also showed that, with an intraperitoneal injection of noggin before I/R, the activation of NF-*κ*B was obviously inhibited. Thus, in future investigations, it will be important to examine the expression of a number of BMP antagonists (noggin, chordin, gremlin, and follistatin) in a rat I/R model.

Additionally, BMP expression regulation is complex. Hypoxia-inducible factor 1 (HIF-1) is one of the master regulators that orchestrate the cellular responses to hypoxia. Recent studies have found that BMP expression is modulated by HIF-1 expression, BMP2 expression was increased in primary chondrocytes under hypoxic conditions, and addition of the HIF-1 activator DFO significantly increased BMP2 expression [39]. Additionally, hypoxia stimulation increased BMP2 mRNA and protein expression levels in osteoblasts via an HIF-1-alpha-dependent mechanism involving the activation of the ILK/Akt and mTOR pathways [40]. Further work is required to better understand the mechanisms guiding the increased BMP expression in the hypoxia and intestinal I/R model.

To the best of our knowledge, the present study is the first to report that BMP2 and BMP4 can directly activate NF-*κ*B, induce the expression of the inflammatory cytokines TNF-a and IL-6 in the intestinal epithelial cells, and decrease the expression of the tight junction protein occludin, which could result in disruption of the intestinal barrier. All of these effects may contribute to the mechanism by which BMP2 and BMP4 mediate intestinal mucosa injury in ischemic reperfusion.
